# CRISPR/Cas9 genome-wide screening identifies LUC7L2 that promotes radioresistance via autophagy in nasopharyngeal carcinoma cells

**DOI:** 10.1038/s41420-021-00783-8

**Published:** 2021-12-14

**Authors:** Lin Shen, Chao Li, Fang Chen, Liangfang Shen, Zhanzhan Li, Na Li

**Affiliations:** 1 Department of Oncology, Xiangya Hospital, Central South University, Changsha, Hunan Province 410008 China; 2 Hunan Key Laboratory of Molecular Precision Medicine, Xiangya Hospital, Central South University, Changsha, Hunan Province 410008 China; 3 National Clinical Research Center for Geriatric Disorders, Xiangya Hospital, Central South University, Changsha, Hunan Province 410008 China

**Keywords:** Head and neck cancer, Cell death, Radiotherapy

## Abstract

Radioresistance emerges as the major obstacle to nasopharyngeal carcinoma (NPC) treatment, further understanding of underlying mechanisms is necessary to overcome the radioresistance and improve the therapeutic effect. In this study, we first identified a candidate radioresistant-related gene *LUC7L2* via CRISPR/Cas9 high-throughput screening and quantitative proteomic approach. Overexpression of LUC7L2 in NPC cells promoted cell viability following exposure to ionizing radiation (IR), while knockdown of LUC7L2 significantly slowed down the DNA replication and impaired cell survival, sensitized NPC-radioresistant cells to IR. Using immunoprecipitation assay, we found SQSTM1, an autophagy receptor, was a potential binding partner of LUC7L2. Down-regulation of LUC7L2 in NPC-radioresistant cells led to reduction of SQSTM1 expression and enhancement of autophagy level. Furthermore, LUC7L2 knockdown in combination with autophagy inhibitor, chloroquine (CQ), resulted in more NPC-radioresistant cell death. Besides, LUC7L2 was obviously distributed in NPC tissues, and high LUC7L2 expression correlated with shorter survival in NPC patients. Our data suggest that LUC7L2 plays a huge part in regulating radioresistance of NPC cells, and serves as a promising therapeutic target in re-sensitizing NPC to radiotherapy.

## Introduction

Nasopharyngeal carcinoma (NPC) is a malignancy tumor that arises in the nasopharynx, located above the oropharynx and hypopharynx. According to the International Agency for Research on Cancer, 133,354 new cases and 80,008 new deaths of NPC were reported in 2020. The majority is geographically localized to East and Southeast Asia, and is likely to show a rising trend annually [1–3]. Since NPC is highly sensitive to ionizing radiation (IR), radiotherapy is the primary treatment to NPC and it induces cell deaths by activating death signals via reactive oxygen species (ROS) generation, DNA damage, and stress response in subcellular organelles. However, a small fraction of cancer cells can escape from been damaged by activating survival pathways, such as DNA repair, unfolded protein response (UPR), and induction of autophagy [4]. Radioresistance remains the main cause resulting in residual or recurrent disease for some patients, leading to treatment failure [5]. Therefore, it is urgent to understand the underlying mechanisms of radioresistance and find an approach to improve the treatment effect.

Multiple experiments have been designed to explore the radioresistant-associated genes or proteins in NPC, for example, at least 2 genes (*gp96* and *GDF15*) were found involved in radioresistance using a cDNA array [6], 4 proteins (14-3-3σ, Maspin, GRP78, and Mn-SOD) were characterized to predict NPC response to radiotherapy by combing two-dimensional electrophoresis and mass spectrometry analysis [7], protein kinase MAPK15 was identified to be a potential regulator of radioresistance [8]. However, no overlap was seen when comparing these data sets, this is possible because of the adoption of different tissues or cell lines, uncovered mechanisms, or limits of technology. In this study, we performed a high-throughput screening towards NPC-radioresistant cell line, by taking advantage of a powerful genome-editing tool, CRISPR/Cas9 system. Ten differential genes were negatively selected based on the copy number analysis. By overlapping with our previous study [8], *LUC7L2* was selected for further investigation. To date, the function of LUC7L2 is not well described and what we know about the protein based on the knowledge from its ortholog LUC7, participating in pre-mRNA splicing [9–11]. Therefore, we explored the functions of LUC7L2 in NPC radioresistance, and our immunoprecipitation assay identified a potential LUC7L2-interacting protein, autophagy receptor SQSTM1.

Autophagy is a conversed process that plays an important role in maintaining cellular homeostasis by degrading and recycling damaged cellular components in response to hostile environment stimuli [12, 13]. Current studies indicate that autophagy is important in the regulation of tumor development, inhibition of autophagy resensitizes the cancer cells to radiotherapy [14**–**17]. An increasing number of clinical trials include autophagy inhibition as part of combination treatment, showing encouraging results, although the mechanism is poorly understood. Our findings revealed LUC7L2 knockdown might promote NPC cell radiosensitivity via elevating autophagy flux, and LUC7L2 could be a potential therapeutical target for NPC radiotherapy.

## Results

### Screen for radioresistant-associated genes/proteins in NPC cells

**Fig. 1:**
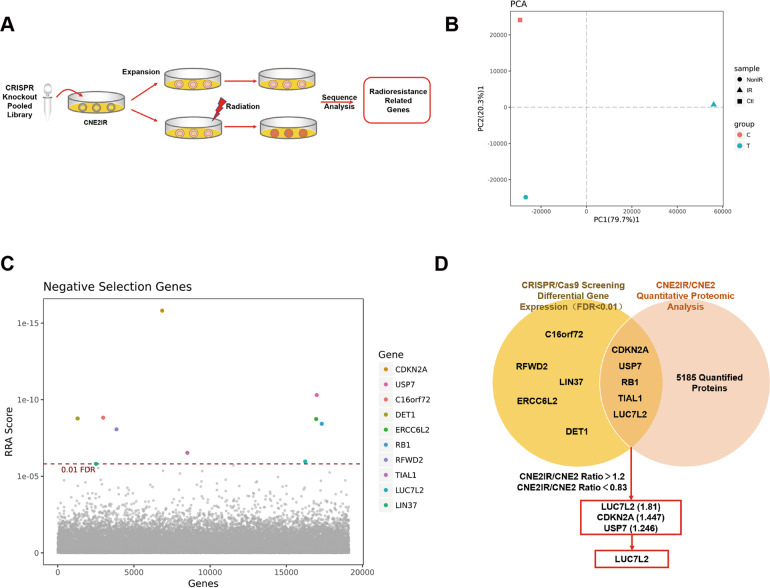
Identification of radioresistant-related genes in NPC cells. **A** Experimental procedure for CRISPR/Cas9 high-throughput screening in CNE2IR cells. **B** PCA analysis on IR/non-IR treated CNE2IR cells. **C** Ten genes were negatively selected according to significant copying number change (FDR < 0.01). **D** *LUC7L2* was identified as a candidate radioresistant-related gene via CRISPR/Cas9 high-throughput screening and quantitative proteomic approach.

NPC-radioresistant cell line CNE2IR was previously constructed and verified to be more resistant to IR compared to CNE2 [7]. To identify the key genes in radioresistant regulation of NPC, we delivered the lentiviral CRISPR knockout pooled library to CNE2IR cells as control (Ctl). Then, infected cells were continuously cultured and divided into two groups, then treated with irradiation (IR) and without irradiation (NonIR). The procedure was shown in Fig. 1A. Three samples were collected and sent to sequencing. Principal Component Analysis (PCA) plot showed that three samples were clearly separated (Fig. 1B). By comparing IR to NonIR group, we did negative selection and found ten genes having extraordinary different copy numbers (FDR < 0.01, Fig. 1C and Table S1). Next, we referred to CNE2IR/CNE2 quantitative proteomic analysis in our previous publications [8, 18], to see if these ten corresponding proteins would also exhibit different expression levels between CNE2IR and CNE2 groups. After comparing these two databases, five genes emerged in the overlap area, including *CDKN2A*, *USP7*, *RB1*, *TIAL1*, and *LUC7L2* (Fig. 1D). Among these genes, three genes (*LUC7L2*, *CDKN2A,* and *USP7*) were selected according to the corresponding protein quantification ratio criteria (CNE2IR/CNE2 fold change > 1.2). CDKN2A and USP7 were reported to function in the radioresistance [19, 20], while LUC7L2 awaits more study. In addition, LUC7L2 got the highest enrichment score (CNE2IR/CNE2 = 1.81). Therefore, we reckoned LUC7L2 might be a potential radioresistant-related target and more investigation would be conducted.

### LUC7L2 is associated with radioresistance of NPC cells

**Fig. 2:**
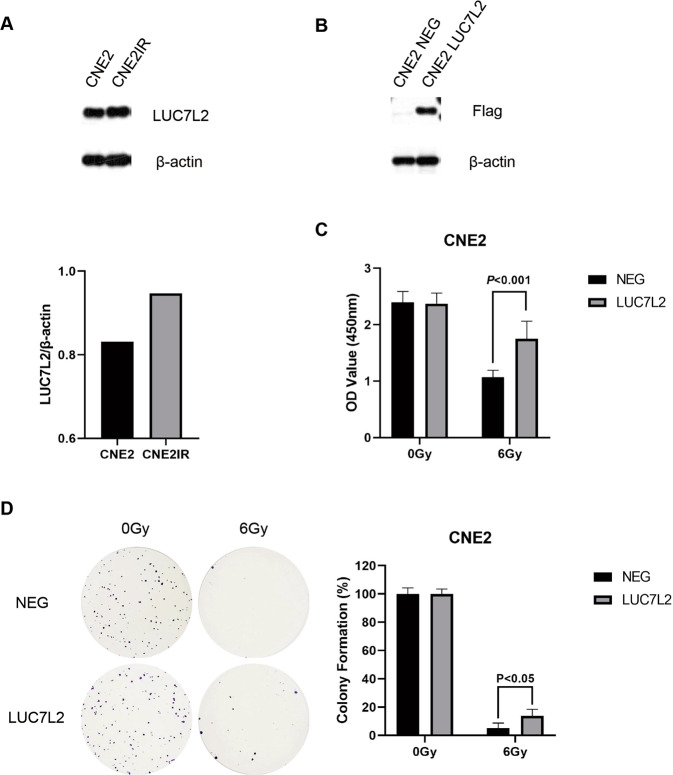
Overexpression of LUC7L2 promoted radioresistance of NPC cells. **A** Comparison of LUC7L2 expression levels in CNE2 and CNE2IR cells. β-actin served as a loading control. **B** Western blot validation on the establishment of CNE2 cell line with stable LUC7L2 overexpression. β-actin served as a loading control. **C** Overexpression of LUC7L2 promoted CNE2 radioresistance. LUC7L2 (6 Gy) vs. NEG (6 Gy), *n* = 5, *P* < 0.001. **D** Clonogenic survival analysis on the radioresistance of CNE2 NEG and CNE2 LUC7L2. The colony formation in CNE2 NEG or CNE2 LUC7L2 without IR was regarded as 100%, respectively. LUC7L2 (6 Gy) vs. NEG (6 Gy), *n* = 3, *P* < 0.05.

Firstly, endogenous expression of LUC7L2 was detected in CNE2 and CNE2IR cells. As shown in Fig. 2A, higher expression level of LUC7L2 was seen in CNE2IR cells. Then, we transfected Flag-LUC7L2 to CNE2 cells, in order to elevate LUC7L2 expression and test if LUC7L2 would help building radioresistance. Western blot result indicated LUC7L2 overexpression in CNE2 cells was successful, using CNE2 NEG as control (Fig. 2B). Thereafter, two cell lines received 0 Gy/6 Gy IR, and CCK8 cell viability assay was conducted after 72 h. Notedly, LUC7L2 upregulation promoted CNE2 radioresistance (Fig. 2C). We have also performed the clonogenic survival assay and obtained the same result (Fig. 2D).

**Fig. 3:**
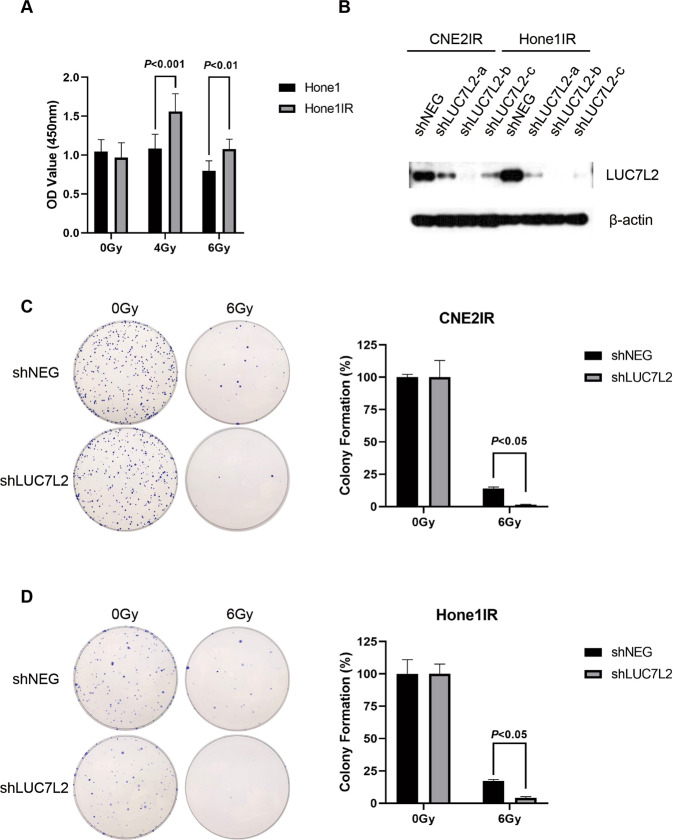
Knockdown of LUC7L2 promoted radiosensitivity of NPC-radioresistant cells. **A** Cell viability assessment on radioresistance of Hone1 and Hone1IR cells. Hone1IR (6 Gy) vs. Hone1 (6 Gy), *n* = 5, *P* < 0.01. **B** Western blot validation on the establishment of CNE2IR and Hone1IR cell lines with stable LUC7L2 knockdown. β-actin served as a loading control. **C** and **D**, Clonogenic survival analysis on the radioresistance of shNEG and shLUC7L2 in NPC cells. The colony formation in shNEG or shLUC7L2 without IR was regarded as 100%, respectively. shLUC7L2 (6 Gy) vs. shNEG (6 Gy), *n* = 3, *P* < 0.05.

**Fig. 4:**
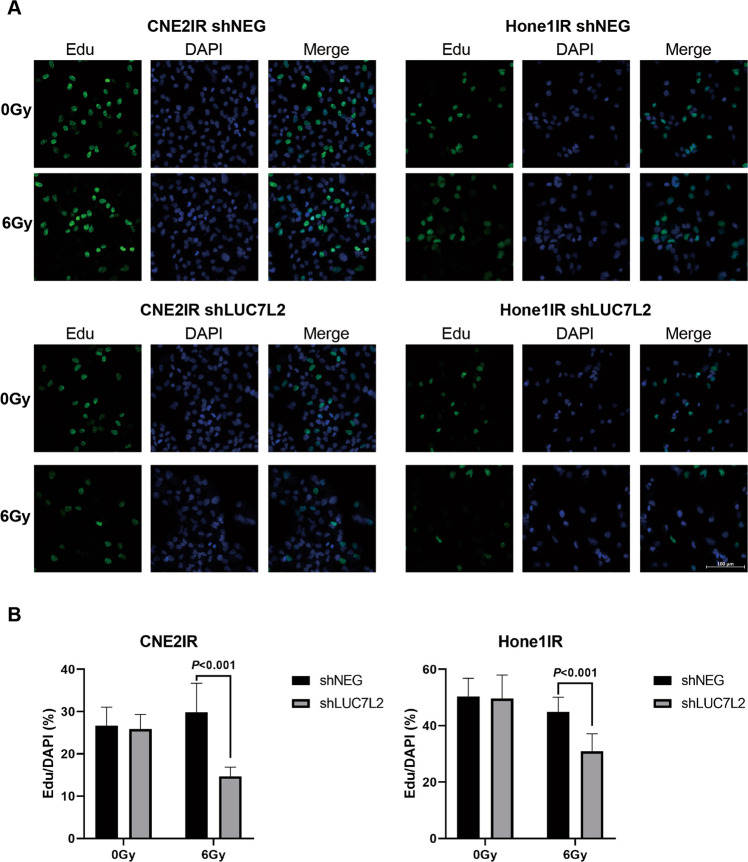
Knockdown of LUC7L2 slowed down the NPC-radioresistant cell replication with IR exposure. **A** CNE2IR and Hone1IR cells (shNEG and shLUC7L2) were treated with 10 μM Edu for 2 h, then stained for Edu (green) and DAPI (blue). Bar: 100 μm. **B** Quantification of Edu/DAPI based on the immunofluorescent images (*n* = 7).

Next, we constructed another NPC-radioresistant cell line Hone1IR by giving 10 Gy irradiation per cycle, up to 6 cycles. Then, the cells received 0 Gy/6 Gy IR, and the viability of cells was measured according to CCK8 assay after 72 h. In contrast of Hone1, Hone1IR exhibited more resistance to 6 Gy IR (Fig. 3A). We constructed NPC LUC7L2 knockdown stable NPC-radioresistant cell lines by lentiviral infection. Our western blot result showed the LUC7L2 knockdown NPC-radioresistant cell lines (shLUC7L2) were successfully established (Fig. 3B), among them, shLUC7L2-b showed highest effectivity. Hence, we chose shLUC7L2-b to proceed the following clonogenic survival experiments. After exposure to 6 Gy IR, shLUC7L2 barely survived in contrast of shNEG (Fig. 3C, D). In addition, we also detected the cell proliferation by Edu staining and quantify Edu-positive cells after exposure to 6 Gy. As shown in Fig. 4A, B, shLUC7L2 cell lines exhibited lower DNA replication activity than shNEG when exposed to 6 Gy. These results indicated that LUC7L2 expression level was closely related to radioresistance.

### Upregulation of LUC7L2 correlated with poor overall survival of NPC patient

**Fig. 5:**
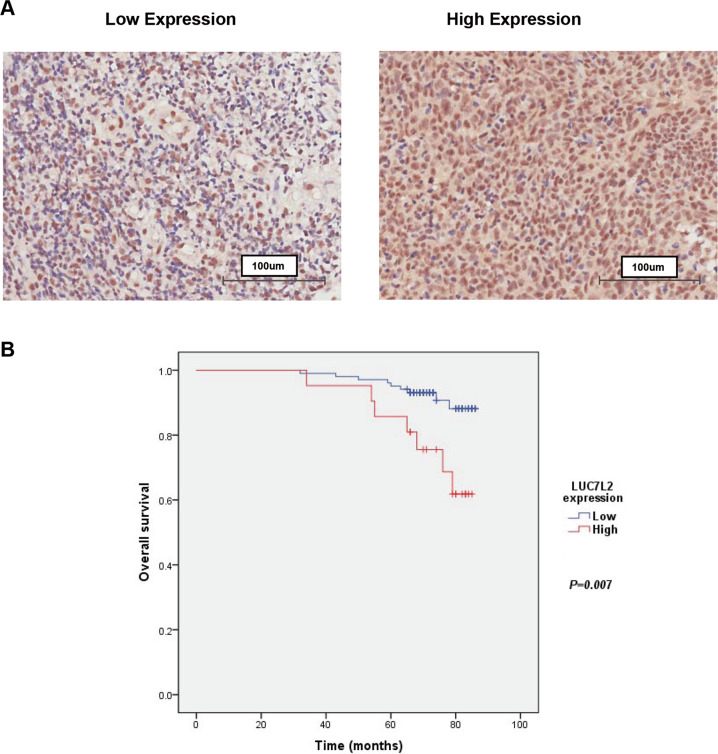
Upregulation of LUC7L2 correlated with poor overall survival of NPC patients. **A** Expression of LUC7L2 in NPC tissues. The LUC7L2 expression level was considered high (>1) or low (≤1) based on the staining intensity. **B** The significant difference in the overall survival was observed in the patients with low LUC7L2 expression and high LUC7L2 expression (*P* = 0.007).

The expression level of LUC7L2 is unknown in NPC patients. To investigate the clinical significance of LUC7L2 in NPC, we performed immunohistochemistry (IHC) staining on a tissue microarray containing 132 NPC patient tissue samples. LUC7L2 was obviously distributed in NPC tissues (Fig. 5A), and its expression intensity score at TNM stage III/IV was higher than stage I/II (*p* = 0.026, Table S2). Kaplan–Meier survival analysis indicated that patients with high LUC7L2 expression had shorter survival than the patients with low LUC7L2 expression (*p* = 0.007, Fig. 5B).

### SQSTM1 might be a potential binding protein of LUC7L2

**Fig. 6:**
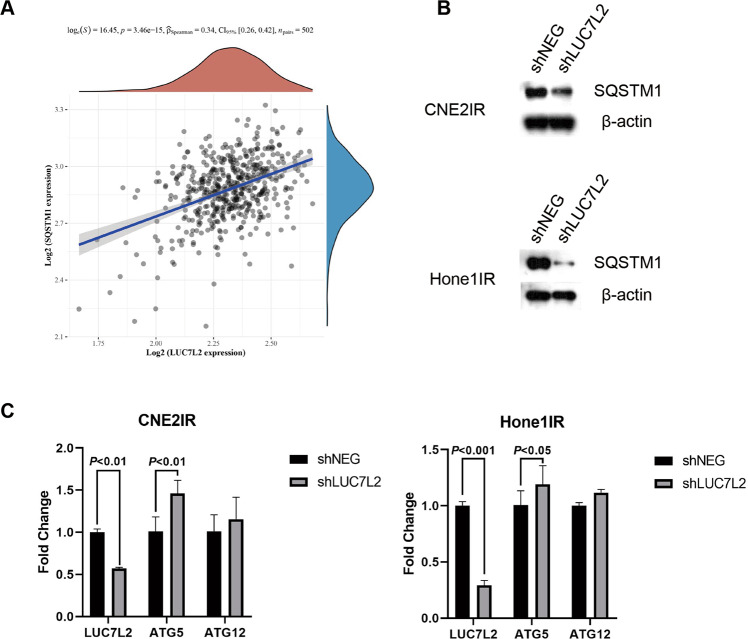
Knockdown of LUC7L2 leads to reduction of SQSTM1 expression. **A** Spearman correlation analysis of LUC7L2 and SQSTM1 in HNSCC patients from the TCGA database. **B** Knockdown of LUC7L2 down-regulates the expression of SQSTM1 in CNE2IR and Hone1IR cells. β-actin served as a loading control. **C** QPCR analysis of LUC7L2, ATG5, and ATG12 in CNE2IR/Hone1IR shNEG and shLUC7L2 cells.

To further explore the possible mechanisms underlying LUC7L2-mediated radioresistant regulation, we performed an Immunoprecipitation assay using the LUC7L2 antibody. Mass spectrography identified 17 potential proteins that might interact with LUC7L2 (Table S3). We noted that SQSTM1, a representative autophagy marker, was on the list. Firstly, we inspected the correlation of the expression of LUC7L2 and SQSTM1 in Head and neck squamous cell carcinomas (HNSCC) using The Cancer Genome Atlas (TCGA) database (*n* = 502). Spearman correlation analysis demonstrated a positive correlation of LUC7L2 and SQSTM1 expression (*p* < 0.001, Fig. 6A). Secondly, we performed western blot to detect the expression of SQSTM1 in NPC-radioresistant cell lines. Our result revealed LUC7L2 knockdown reduced expression of SQSTM1 (Fig. 6B). Since SQSTM1 played an important role in autophagy, we wondered if there were other autophagy-related genes involved. Quantitative real-time PCR (qPCR) was used to detect the mRNA levels of autophagy-related genes, and we found ATG5 was upregulated in shLUC7L2 cell lines (Fig. 6C). ATG5-ATG12 conjugate acted as an E3 ligase for LC3 and was regarded to be essential for LC3 lipidation [21]. We postulated LUC7L2 might get involved in autophagy through regulating SQSTM1 expression.

### Knockdown of LUC7L2 increased autophagy level in NPC-radioresistant cells

**Fig. 7:**
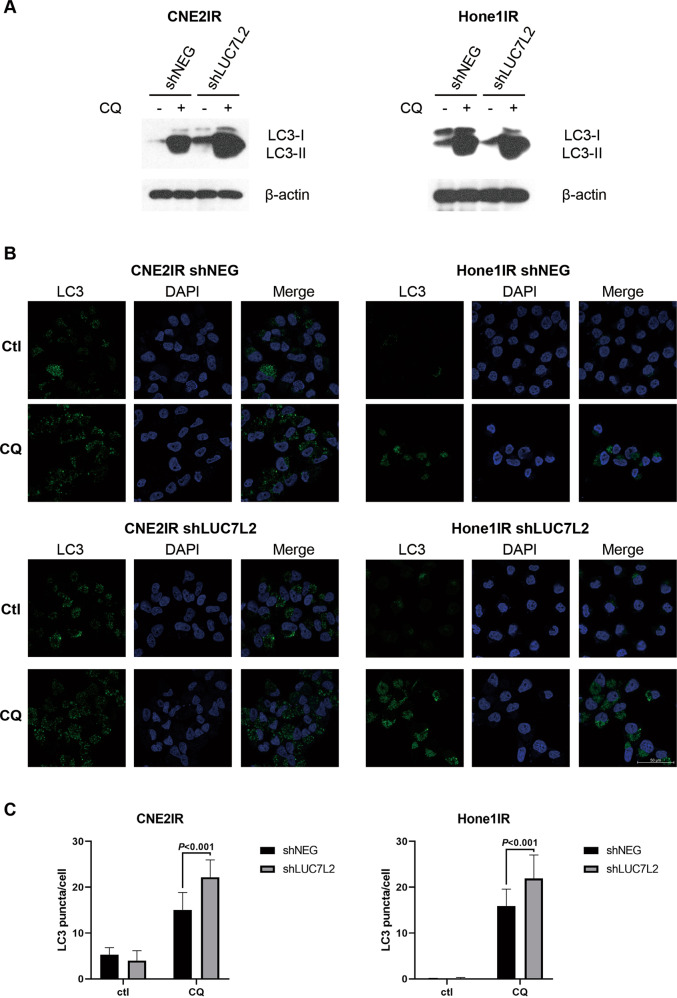
Knockdown of LUC7L2 increased autophagy level in NPC-radioresistant cells. **A** Knockdown of LUC7L2 in CNE2IR increased LC3-II expression level after 24 h treatment of 50 μM CQ. Knockdown of LUC7L2 in Hone1IR slightly influenced the LC3-II level. β-actin served as a loading control. **B** CNE2IR and Hone1IR cells (shNEG and shLUC7L2) were treated with 50 μM CQ for 24 h, then stained for LC3 (green) and DAPI (blue). Bar: 50 μm. **C** Quantification of LC3 puncta based on the immunofluorescent images (*n* = 8).

To better illuminate the possible connection of LUC7L2 and autophagy, we monitored the autophagy flux by detecting LC3-II expression in NPC-radioresistant cell lines. Endogenous LC3 is detected as two bands by immunoblotting, LC3-I and LC3-II. LC3-I is cytosolic, it is cleaved upon autophagy induction and conjugated to phosphatidylethanolamine (PE) to become LC3-II, which presents on autophagosomal membranes. Autophagosomes fuse with lysosomes to degrade their contents, LC3-II is also degraded and recycled to LC3-I. Therefore, the level of LC3-II is generally indicative of autophagy induction. While the amount of LC3-II alone at a certain point does not always indicate the autophagy flux because of its own degradation, it is better to measure the LC3-II amount with and without lysosomal protease inhibitors [22], such as chloroquine (CQ), which blocks autophagosome-lysosome fusion. We treated NPC-radioresistant shNEG and shLUC7L2 cells with or without 50 μM CQ for 24 h, and then performed western blot. We found LC3-II expression was enhanced in shLUC7L2 cells compared to shNEG upon CQ treatment in CNE2IR cells, but not much difference was seen in Hone1IR cells (Fig. 7A). Next, we checked the autophagy level by performing LC3 puncta formation assay. Endogenous LC3 was detected using immunofluorescence microscopy after 24 h CQ treatment. As shown in Fig. 7B, Hone1IR shNEG cells seemed to have much less LC3 puncta than that of CNE2IR shNEG cells, while the CQ treatment augmented the LC3 puncta in both shNEG cells separately. In addition, more LC3 puncta were observed in shLUC7L2 cells than that of shNEG cells upon CQ treatment (*P* < 0.001, Fig. 7C). We believed that knockdown of LUC7L2 increased the autophagy level in NPC-radioresistant cells.

### Enhancement of autophagy level mediated by LUC7L2 led to more NPC-radioresistant cell death

To our knowledge, the role of autophagy in cancer development is controversial, because either enhancement or reduction of autophagy might have good clinical outcomes in different cancers or clinical stages [23]. To further analyze the effect of LUC7L2 related autophagy effect in NPC-radioresistant cells, we utilized flow cytometry to observe the apoptosis status in LUC7L2-deficient cells. NPC-radioresistant shNEG and shLUC7L2 cells were treated with or without 50 μM and 100 μM CQ for 24 h, and then stained with Propidium lodide (PI) and Annexin-V FITC.

**Fig. 8:**
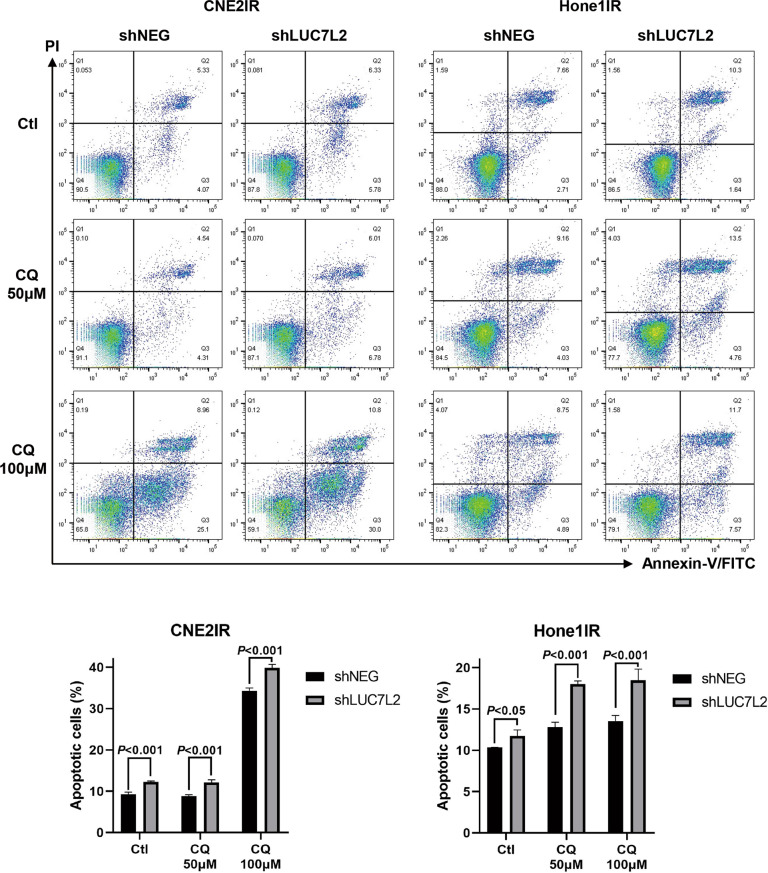
Apoptosis analysis by flow cytometry. CNE2IR and Hone1IR cells (shNEG and shLUC7L2) were treated with 0/50/100 μM CQ for 24 h, then stained with PI and Annexin-V FITC.

The percentage of apoptotic cells was calculated (Fig. 8). More cell deaths were observed in shLUC7L2 cells than shNEG cells regardless of CQ treatment, however, the difference of apoptotic cell percentage between shNEG and shLUC7L2 extended from ~3.0% (CNE2IR, Ctl, and CQ 50 μM) to 5.6% (CNE2IR, CQ 100 μM), and from 1.4% (Hone1IR, Ctl) to ~5.0% (Hone1IR, CQ 50 μM and CQ 100 μM) respectively. This result revealed that LUC7L2 deficiency led to NPC-radioresistant cell death, and this effect could be magnified along with the addition of autophagy inhibitor.

## Discussion

With introduction of high-throughput technologies in cancer research, it is becoming easier and more productive to obtain a vast number of data providing us more insights on cancer development, prevention, diagnosis, and treatment [24]. For example, the application of quantitative proteomic techniques and CRISPR/Cas9 high-throughput screening assists us to discover novel diagnostic or prognostic biomarkers, and identify potential therapeutical targets [25, 26]. However, digging out the valuable information through numerous data might be more challenge and time-consuming.

In this study, we have first taken advantage of CRISPR/Cas9 high-throughput screening to negatively select essential genes that might be related to radioresistance in NPC. Ten genes were selected according to significant copying number change (FDR < 0.01). Then we narrowed down the gene list by visiting our previous study elaborating potential radioresistant-related proteins via quantitative proteomic approach [8]. Five corresponding proteins of ten were successfully quantified, and three proteins, LUC7L2, CDKN2A, and USP7, were picked based on the protein quantification ratio criteria (CNE2IR/CNE2 fold change>1.2). CDKN2A gene is hypo-methylated in radioresistant NPC cells [19], and USP7-depleted cells were more sensitive to IR [20], while the radioresistant-associated function of LUC7L2 has not been reported. Another reason we focused on LUC7L2 is due to the highest score (CNE2IR/CNE2 ratio = 1.81). LUC7L2 is RNA binding protein that has not been well characterized in the past years. Our findings depict the radioresistance role of LUC7L2, that knockdown of LUC7L2 will significantly improve radiosensitivity in NPC cells.

To better understand the underlying mechanisms, we started with protein-protein interacting network exploration by means of immunoprecipitation assay. Surprisingly, we found an autophagy receptor protein, SQSTM1, which might be interacting with LUC7L2. It is interesting because *LUC7L2* is regarded as an RNA-splicing gene and involved in mRNA transcript [11, 27], autophagy-related function of LUC7L2 has not been reported. Many studies suggested autophagy play a huge part in NPC radioresistance. Latent membrane protein 1 (LMP1), an essential protein involved in NPC pathogenesis, contributes to radioresistance by enhancing autophagy via upregulating the expression of BCL2/adenovirus E1B 19 kDa protein-interacting protein 3 (BNIP3) [28]. Wnt3a induces autophagy and sensitized squamous cell carcinoma of the head and neck (SCCHN) to IR in vitro and in vivo [17]. Autophagy inhibitor CQ, the only clinically available drug to inhibit autophagy [23], sensitizes nasopharyngeal carcinoma cells to IR [29]. In our study, we discovered a new mechanism that LUC7L2 knockdown led to reduction of SQSTM1 expression and upregulated ATG5 mRNA level, along with the elevated autophagy flux. Moreover, an increasing number of NPC-radioresistant dead cells were observed by a combination of LUC7L2 knockdown and autophagy inhibitor CQ.

Recent researches have shown that the deficiency of *LUC7L2* results in aberrant splicing of transcripts which probably contribute to the pathogenesis of myelodysplastic syndromes (MDS) [30]. Depletion of *LUC7L2* loci is more prevalent in high-risk MDS than in low-risk MDS, and low LUC7L2 expression correlates with significantly shorter survival in primary acute myeloid leukemia (pAML) patients [31]. In this study, we explored the LUC7L2 expression in NPC patient tissue samples. Different from MDS patients, high LUC7L2 expression correlated with a shorter survival in NPC patients. It was possible that cells with high expression of LUC7L2 tended to overcome the radiotherapy, leading to more cancer cell survival. While more studies are needed to elaborate on the specific mechanisms.

In summary, LUC7L2 was identified by means of CRISPR/Cas9 high-throughput screening and quantitative proteomic approach which is associated with radioresistance of NPC cells. LUC7L2 knockdown potentially led to more NPC-radioresistant cell death by decreasing expression of SQSTM1 and increasing autophagy flux. Targeting LUC7L2 might be useful in sensitizing NPC to radiotherapy.

## Material and methods

### Cell lines

Human NPC cell line Hone1, CNE2, and its radioresistant subline CNE2IR, were well-characterized [7] and kindly provided by Dr. Zhiqiang Xiao (Key Laboratory of Cancer Proteomics of Chinese Ministry of Health, Central South University, Changsha, China). These NPC cell lines were cultured in Dulbecco’s modified Eagle’s medium (DMEM, Hyclone) supplemented with 10% FBS at 37 °C in 5% CO_2_.

Hone1 cells were seeded at a density of 1 × 10^5^ per T25 flask in DMEM with 10% FBS and cultured in the incubator. On the 2nd day, cells received 10 Gy dose of irradiation. After treatment, the majority of cells started to die, and it took 1–2 weeks for the survival cells to recover. Until the cell density reached ~1 × 10^5^ per T25 flask, another 10 Gy was given to the 1st generation of the subclone cells. Up to 6 generations of subclone Hone1 cells were produced, and we defined the 6th generation of cells which received a total of 60 Gy as the radioresistant subclone cell line and named for Hone1IR. Experiments were performed with Hone1IR cells within 4–10 passages after the termination of irradiation.

### Plasmids and reagents

Lentiviral LUC7L2 expression plasmid and control plasmid pReceiver-Lv242, lentiviral LUC7L2 shRNA-a, b, c expression plasmid, and control plasmid psi-LVRU6P were purchased from GeneCopoeia (MD, USA). Chloroquine (HY-17589) was purchased from MedChemExpress (NJ, USA).

### Genome-scale CRISPR/Cas9 high-throughput screening

Human CRISPR Knockout Pooled Library (Brunello, 1 × 10^8^TU) was purchased from Beijing Genomtech Co. (Beijing, China). On the 1st day, ~2 × 10^8^ CNE2IR cells were suspended in 40 mL (5 × 10^6^/mL) DMEM with 10% FBS, mixed with lentiviral library and 8 μg/mL polybrene, gently inverted, and then seeded to 13 T225 flasks (~1.5 × 10^7^/flask). Another 1.5 × 10^7^ cells without lentiviral library were seeded in 1 T225 flask as control. After 48 h culture, puromycin (2 μg/mL) was added to all flasks for selection. Cells were cultured and passaged until Day9, control cells without lentiviral library were dead due to 2 μg/mL puromycin incorporation, all the other infected cells were counted and divided into 3 groups, ~6 × 10^7^ cells were washed twice using PBS, and pelleted to store at −80 °C as sample Ctl, the 2nd group of ~6 × 10^7^ cells were seeded to 7 T225 flasks (~8.5 × 10^6^/flask) and continued to be cultured (sample: NonIR), the 3rd group of ~6 × 10^7^ cells were seeded to 7 T225 flasks and received irradiation (sample: IR; 6 Gy each cycle, up to 4 cycles). After 2 months, 3 groups of cells were collected and sent to sequencing analysis by Beijing Genomtech Co.

### Establishment of NPC cell lines with stable LUC7L2 overexpression and knockdown

Lentiviral pReceiver-Lv242 vector expressing LUC7L2 or control NEG, and a lentiviral psi-LVRU6P vector expressing LUC7L2 shRNA-a, b, c or control shNEG were co-transfected with packaging plasmids (Lenti-Pac HIV Expression Packaging Kit, GeneCopoeia) to 293FT packaging cells. Lentiviral particles were collected after 48 h, and concentrated using Lenti-Pac Lentivirus Concentration Solution (GeneCopoeia). Afterwards, cells were infected by the lentiviral particles separately and then selected using 2 μg/mL puromycin for 2 weeks. NPC cells with stable overexpression or knockdown of LUC7L2 and NEG were constructed.

### Clonogenic survival assay

NPC cells were seeded at a density of 5 × 10^2^ per 6 cm dish and irradiated with 6 Gy at room temperature at 300 cGy/min with a linear accelerator (2100EX, Varian, USA). After 12 days, the surviving colonies (defined as a colony with >50 cells) were stained with 0.5% crystal violet and counted. Three independent experiments were done.

### Cell viability analysis

NPC cells were seeded at 96w plate (5 × 10^3^ per well) and irradiated with 6 Gy. After 72 h, cells were incubated with CCK8 (Cell Counting Kit-8, Dojindo, Japan) for 1 h at 37 °C in 5% CO_2_, then the absorbance at 450 nm was measured with the microplate reader. Three independent experiments were done.

### Edu staining proliferation assay

Cells were irradiated with 0 Gy/6 Gy. After 48 h, cells were incubated with Edu for another 2 h, and then fixed with 4% PFA for 15 min, rinsed in PBS for three times, and permeabilized in PBS with addition of 0.3% Triton X-100, rinsed in PBS, and followed the procedure according to the Edu-488 cell proliferation detection kit (C0071S, Beyotime Biotechnology, Shanghai, China). Images were collected on the ZEISS LSM880 confocal system.

### Immunoprecipitation and mass spectrometry analysis

CNE2IR cells were harvested and suspended in cell lysis buffer on ice, incorporated with Protein A + G agarose (P2012, Beyotime Biotechnology) and rabbit IgG (A7016, Beyotime Biotechnology) to remove non-specific binding. After 2 h incubation on ice, the agarose was discarded and the supernatant was reserved to incubate with or without LUC7L2 antibody at 4 °C overnight. On the 2nd day, the new agarose was added to the supernatant for further 3 h incubation and then washed with cold lysis buffer five times. The agarose was boiled in an SDS-PAGE sample buffer followed by SDS-PAGE. The gel slices were cut and sent to Jingjie PTM Biolab (Hangzhou, China) for mass spectrometry analysis.

### Western blot

Protein lysates (15 μg) were separated by 12% SDS-PAGE and transferred to PVDF membranes (Amersham Biosciences, Uppsala, Sweden). Blots were blocked with 5% non-fat dry milk for 1 h at room temperature and then incubated with 1:1000 dilutions of anti-LUC7L2 antibody (ab241356, Abcam), anti-LC3 antibody (#12741, Cell Signaling Technology), and anti-FLAG antibody (#2368, Cell Signaling Technology), 1:10,000 dilution of anti-SQSTM1 antibody (ab109012, Abcam), for 2 h at room temperature, followed by incubation with 1:10,000 dilutions of peroxidase-conjugated goat anti-rabbit IgG (111-035-003, Jackson ImmunoResearch), or peroxidase-conjugated goat anti-mouse IgG (115-035-003, Jackson ImmunoResearch), for 1 h at room temperature. The signals were visualized with an enhanced chemiluminescence detection reagent (Abcam). β-Actin was detected simultaneously using 1:1000 dilutions of anti-β-actin antibody (AF0003, Beyotime Biotechnology) as a loading control.

### RNA extraction and quantitation real-time PCR

Cells were collected and washed twice with PBS. RNA extraction and cDNA synthesis were performed using RNeasy micro kit (Qiagen) and SureScript™ First-Strand cDNA Synthesis Kit (GeneCopoeia) respectively. QPCR Primers were ordered from GeneCopoeia. QPCR experiments were conducted using SYBR Green (QST-100, Toroivd Technology Company, Shanghai, China) method, and detected by the Applied Biosystems QuantStudio 3 Real-Time PCR system. Three independent experiments were done.

### Spearman’s correlation analysis of LUC7L2 and SQSTM1

mRNA-seq data of LUC7L2 and SQSTM1 were obtained in HNSCC using TCGA database (*n* = 502). LUC7L2-SQSTM1 correlation map was drawn by the R software package “ggstatsplot”.

### Immunofluorescence microscopy

Cells were seeded onto the sterile 12 mm coverslips in 24w plate. Cultured cells were fixed in 4% PFA, rinsed in PBS for three times, and permeabilized in PBS with the addition of 0.1% Triton X-100, then washed with PBS and blocked in PBS with the addition of 1% BSA. Next, the cells were incubated with 1:100 dilution of anti-LC3 antibody (#12741, Cell Signaling Technology) at 4 °C overnight. On the 2nd day, the cells were rinsed three times in PBST (0.05% Tween in PBS), and incubated with 1:500 dilution of fluorescent-labeled secondary antibodies, alexa fluor 488-conjugated donkey anti-rabbit IgG (711-545-152, Jackson ImmunoResearch) in dark for 1 h. Then, the cells were washed with PBST before mounting in antifade mounting medium with DAPI (H-2000, VECTASHIELD, CA, USA). Images were collected on the ZEISS LSM880 confocal system.

### Immunohistochemistry

IHC assays were performed as previously described using anti-LUC7L2 antibodies (1:1000) [32]. A human tissue microarray containing 132 NPC patient tissue samples and the associated clinicopathological information was purchased from Shanghai OUTDO Biotech Co. (Shanghai, China). The experiment was approved by Committee on the Shanghai Outdo Biotech Company (Project number: T20-1472). LUC7L2 immunostaining was scored based on the extent of positive cell staining (0, 0%; 1, 1–25%; 2, 26–50%; 3, 51–75%; and 4, 76–100%) and the staining intensity (0, no staining; 1, slight staining; 2, moderate staining; and 3, strong staining). The LUC7L2 expression level was considered high (>1) or low (≤1) based on the staining intensity.

### Apoptosis analysis by flow cytometry

Cell apoptosis was determined by flow cytometry using FITC Annexin V Apoptosis Detection Kit with PI (640914, Biolegend, CA, USA). Briefly, cells were washed twice with PBS, and resuspended. Then, 100 μL cell suspension was transferred to 1.5 ml EP tubes and stained with FITC Annexin V and PI for 15 min at room temperature, followed by adding 400 μL binding buffer and analyzed by flow cytometry machine. Three independent experiments were done.

### Statistical analysis

Two-tailed Student’s *t* tests were performed using GraphPad Prism 8.0. Survival analysis was performed using the Kaplan–Meier method with the log-rank test by IBM SPSS Statistics 23. Differences at *p* < 0.05 were considered statistically significant.

## Supplementary information

Table S1

Table S2

Table S3

## Data Availability

All data generated or analyzed during this study are included in this published article and its supplementary information files.
