# Supplementary material for: CRISPR/Cas9 genome-wide screening identifies LUC7L2 that promotes radioresistance via autophagy in nasopharyngeal carcinoma cells
Source: Cell Death Discov. 2021 Dec 14;7:392. doi: 10.1038/s41420-021-00783-8 (PMC8671510; doi:10.1038/s41420-021-00783-8)
Supplement: Supplementary file 2 — Table S2 [file 41420_2021_783_MOESM2_ESM.docx]

**Table S2. Correlation between LUC7L2 expression and clinicopathological characteristics.**

|  | **Variables** | **LUC7L2 Expression** | | **Total** | **χ2** | **P value** |
| --- | --- | --- | --- | --- | --- | --- |
|  |  | **Low** | **High** |  |  |  |
| **Age (year)** |  |  |  |  | 3.252 | 0.071 |
|  | ≤50 | 56 | 16 | 72 |  |  |
|  | >50 | 46 | 5 | 51 |  |  |
| **Sex** |  |  |  |  | 0.005 | 0.946 |
|  | Female | 25 | 5 | 30 |  |  |
|  | male | 77 | 16 | 93 |  |  |
| **TNM stage** |  |  |  |  | 4.936 | 0.026 |
|  | Ι/II | 61 | 7 | 68 |  |  |
|  | III/IV | 41 | 14 | 55 |  |  |
| **Metastatic-NPC** |  |  |  |  | 2.259 | 0.133 |
|  | NO | 31 | 3 | 34 |  |  |
|  | YES | 71 | 18 | 89 |  |  |
