# Supplementary material for: CRISPR/Cas9 genome-wide screening identifies LUC7L2 that promotes radioresistance via autophagy in nasopharyngeal carcinoma cells
Source: Cell Death Discov. 2021 Dec 14;7:392. doi: 10.1038/s41420-021-00783-8 (PMC8671510; doi:10.1038/s41420-021-00783-8)
Supplement: Supplementary file 3 — Table S3 [file 41420_2021_783_MOESM3_ESM.docx]

**Table S3. Potential binding proteins to LUC7L2.**

| **Accession** | **Protein Names** | **Gene Names** |
| --- | --- | --- |
| Q14247 | Src substrate cortactin | CTTN |
| Q13501 | Sequestosome-1 | SQSTM1 |
| P08727 | Keratin, type I cytoskeletal 19 | KRT19 |
| Q96AQ8 | Mitochondrial calcium uniporter regulator 1 | MCUR1 |
| P52272 | Heterogeneous nuclear ribonucleoprotein M | HNRNPM |
| P27348 | 14-3-3 protein theta | YWHAQ |
| Q96HS1 | Serine/threonine-protein phosphatase PGAM5, mitochondrial | PGAM5 |
| P62249 | 40S ribosomal protein S16 | RPS16 |
| Q02543 | 60S ribosomal protein L18a | RPL18A |
| Q07020 | 60S ribosomal protein L18 | RPL18 |
| P61353 | 60S ribosomal protein L27 | RPL27 |
| P00966 | Argininosuccinate synthase | ASS1 |
| Q9H361 | Polyadenylate-binding protein 3 | PABPC3 |
| Q8NB90 | ATPase family protein 2 homolog | SPATA5 |
| P46781 | 40S ribosomal protein S9 | RPS9 |
| Q9UBC2 | Epidermal growth factor receptor substrate 15-like 1 | EPS15L1 |
| P62266 | 40S ribosomal protein S23 | RPS23 |
